# Phenotype to genotype using forward-genetic Mu-seq for identification and functional classification of maize mutants

**DOI:** 10.3389/fpls.2013.00545

**Published:** 2014-01-07

**Authors:** Charles T. Hunter, Masaharu Suzuki, Jonathan Saunders, Shan Wu, Alexander Tasi, Donald R. McCarty, Karen E. Koch

**Affiliations:** Plant Molecular and Cellular Biology Program, Institute of Food and Agricultural Sciences, Horticultural Sciences, University of FloridaGainesville, FL, USA

**Keywords:** forward genetics, mutator, transposon, sequencing, Mu-Seq, UniformMu

## Abstract

In pursuing our long-term goals of identifying causal genes for mutant phenotypes in maize, we have developed a new, phenotype-to-genotype approach for transposon-based resources, and used this to identify candidate genes that co-segregate with visible kernel mutants. The strategy incorporates a redesigned Mu-seq protocol (sequence-based, transposon mapping) for high-throughput identification of individual plants carrying Mu insertions. Forward-genetic Mu-seq also involves a genetic pipeline for generating families that segregate for mutants of interest, and grid designs for concurrent analysis of genotypes in multiple families. Critically, this approach not only eliminates gene-specific PCR genotyping, but also profiles all Mu-insertions in hundreds of individuals simultaneously. Here, we employ this scalable approach to study 12 families that showed Mendelian segregation of visible seed mutants. These families were analyzed in parallel, and 7 showed clear co-segregation between the selected phenotype and a Mu insertion in a specific gene. Results were confirmed by PCR. Mutant genes that associated with kernel phenotypes include those encoding: a new allele of Whirly1 (a transcription factor with high affinity for organellar and single-stranded DNA), a predicted splicing factor with a KH domain, a small protein with unknown function, a putative mitochondrial transcription-termination factor, and three proteins with pentatricopeptide repeat domains (predicted mitochondrial). Identification of such associations allows mutants to be prioritized for subsequent research based on their functional annotations. Forward-genetic Mu-seq also allows a systematic dissection of mutant classes with similar phenotypes. In the present work, a high proportion of kernel phenotypes were associated with mutations affecting organellar gene transcription and processing, highlighting the importance and non-redundance of genes controlling these aspects of seed development.

## Introduction

Strategies for inducing and recovering insertional mutations are an important foundation of functional genomics. Model plant species for which there are public collections of mutants include Arabidopsis (Parinov et al., 1999; Tissier et al., 1999; Galbiati et al., 2000; Samson et al., 2002; Sessions et al., 2002; Alonso et al., 2003; Rosso et al., 2003; Kuromori et al., 2004; Woody et al., 2007), rice (Miyao et al., 2003; Jeong et al., 2006; Zhang et al., 2006; Hsing et al., 2007; Miyao et al., 2007; Krishnan et al., 2009), and maize (Bensen et al., 1995; Meeley and Briggs, 1995; Raizada and Walbot, 2000; Walbot, 2000; Cowperthwaite et al., 2002; May et al., 2003; Slotkin et al., 2003; Ahern et al., 2009; Vollbrecht et al., 2010; McCarty et al., 2013b). These reverse-genetic resources have proven invaluable to investigations of gene functions. In particular, the UniformMu maize population (McCarty et al., 2005) is a public resource for insertional mutagenesis based on the Robertson's Mutator (Mu) transposon (Robertson, 1978). The resource currently comprises over 8000 lines and 35,000 genic insertions that are accessible online (maizegdb.org; McCarty et al., 2005; Settles et al., 2007; McCarty et al., 2013a,b).

Use of insertion-based resources, whether for forward or reverse genetics, requires accurate and reliable approaches for tracking mutations. Typically, a small number of segregating F2 individuals from a UniformMu maize family (or from a similar resource) are genotyped by PCR for the presence/absence of a particular Mu insertion. Gene-specific PCR primers are required, as well as individual DNA extractions for each plant, and prior knowledge of sites for candidate gene insertions. While this approach has been employed effectively over the last two decades (Settles et al., 2007; Hunter et al., 2012; McCarty et al., 2013a,b), development and optimization of reliable, gene-specific, PCR-genotyping assays can be time-, labor-, and resource-intensive. The challenges include PCR-recalcitrant sequences, false positives, and inconsistent results. The value of a more efficient approach is especially clear when large numbers of insertions are being followed in multiple lines, particularly for high-copy transposon systems like that of Mutator.

Construction of the UniformMu resource has fostered development of high-throughput sequencing strategies that enable efficient mapping of new Mu insertions in the maize genome (Settles et al., 2007; McCarty et al., 2013a,b). The common goal of these approaches has been to amplify and sequence genomic DNA immediately flanking germinal Mu insertion sites in large numbers of maize plants. The most advanced of these sequencing methods, Mu-seq, specifically amplifies regions of DNA that extend from the highly-conserved, terminal inverted repeat (TIR) sequence of the Mu element into the immediately-adjacent sequence of the host genome (McCarty et al., 2013b). The key feature of this technology lies in its capacity to map thousands of Mu insertions precisely, and to do so concurrently in a large number of maize lines. Sequencing “reads” resulting from Mu-seq analysis have short regions of Mu-TIR sequence that confirm their Mu-anchored origin, followed by specific host sequences that flank Mu insertion sites. A comparison of these reads to the maize genome allows insertion sites to be mapped. Multiplexing is enabled by inclusion of a 4-base, sample-specific barcode that allows up to 64 DNA libraries to be sequenced in the same flow cell of an Illumina sequencer. The reliability of this method has been demonstrated during construction of the UniformMu resource for reverse genetics (McCarty et al., 2013b) and has been used to map novel Mu insertions to over 9000 specific maize lines.

Here we present forward-genetic Mu-seq, a strategy designed for linking phenotypes with their causal, transposon mutations by taking high-throughput profiling of transposon insertions to the resolution of individual plants within arrangements of multiple, segregating families. The approach includes the genetic analyses involved in generating these families, and grid designs that incorporate genotype and phenotype. We use forward-genetic Mu-seq to simultantously track the Mu elements in each plant and family and compare their presence with the expression of a given phenotype. Results allow co-segregation analyses to be conducted on a much larger scale (few to many concurrent families) and with vastly improved accuracy, time input, and cost effectiveness over PCR alone. In addition to the efficacy of this approach, we show the outcome of its use; a set of seed mutants linked with putative, causal genes. These data not only allow prioritization of mutants for subsequent study based on gene annotations, but also demonstrate how reverse-genetic Mu-seq can be used to dissect a specific developmental pathway. We analyze the selected seed mutants with the goal of determining the specific developmental or physiological processes impaired in these similar mutants. Results presented here highlight the critical role of nuclear-encoded proteins that influence expression and transcript processing of organellar genes during maize kernel development. The capacity for systematic analysis of multiple mutants is especially important for genetic dissection of complex processes, such as seed formation, that involve a large number of loci.

## Materials and methods

### Plant material, mating strategy, and phenotyping

All maize lines used were selected from the publicly-available, UniformMu collection, based on the presence of a kernel phenotype. Plants were grown under field conditions at the UF-Plant Science Research Unit at Citra, FL and self-pollinated to check for heritability, as well as back-crossed to the wildtype, W22-inbred to generate F2 segregating progeny. Segregating progeny were also grown under field conditions and self-pollinated. Prior to pollination, leaf samples were collected for later use in construction of forward-genetics Mu-seq grids. Ears from self-pollinated plants were scored for presence/absence of seed phenotypes. Kernels were imaged using a Leica MZ 125 dissecting microscope with an attached camera (Diagnostic Instruments, model 2.3.1).

### Grid construction and DNA extraction

**Figure 1:**
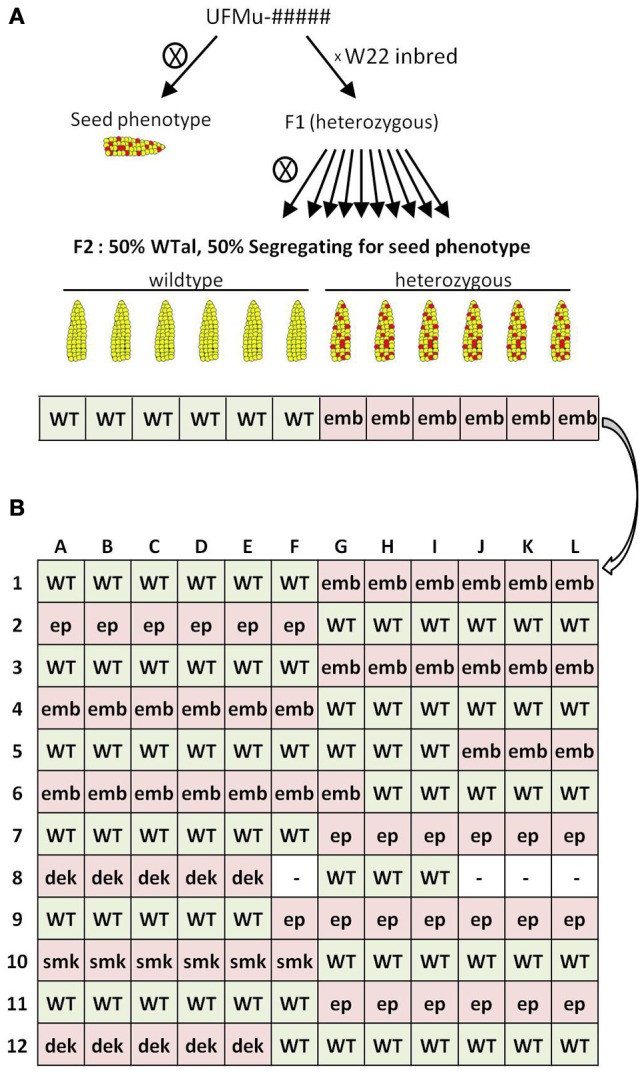
**Genetic strategy and grid design for forward-genetics Mu-seq. (A)** UniformMu families segregating for seed phenotypes were selected for inclusion in the forward-genetics Mu-seq grid. For selected families, individuals were backcrossed to the W22 inbred, followed by selfing of the F1 progeny. Resulting ears were examined for the phenotype of interest, and scored as positive or negative. The expected ratio of ears with and without visible phenotypes was 50%. **(B)** Leaf samples from mothers of the phenotyped ears were pooled according to the diagramed grid design, such that pools 1 through 12 represented twelve individuals from a single family, and pools A through L represented a single individual from each family. DNA was extracted from the resulting 24 pools of leaf samples, sequencing libraries were constructed with each pool being assigned a specific key-code identifier, and Mu flanks were sequenced using a single Illumina flow cell. Seed phenotype abbreviations: embryo lethal (emb), empty pericarp (ep), small kernel (smk), defective kernel (dek).

Figure 1B shows the construction strategy for a 12-by-12, forward-genetics Mu-seq grid of kernel mutants. Leaf samples were pooled based on family and plant number, so that individuals were represented once in each axis. Each of the 24 pools contained 12 leaf samples, and genomic DNA was extracted from each pool. Samples (about 10 g) were ground in liquid nitrogen and frozen powder was added to 20 mL of extraction buffer (42% Urea, 6% NaCl [5 M], 5% Tris-HCl [pH 8.0], 4% EDTA [0.5 M], 1% Sarkosyl [Fisher Lot # 101874]). Samples were allowed to thaw before addition of 5 mL phenol (Fisher Lot # 116885) and 5 mL chloroform solution (24 part chloroform [Fisher Lot # 066906], 1 part 3-methylbutanol [Sigma 19392-500ML]). Solutions were thoroughly mixed and transferred to 50-mL, Phase Lock gel tubes (Eppendorf Cat # 06-443-18) for centrifugation at 4000 rpm for 5 min in a Thermo Forma swinging-bucket centrifuge (model 5530 1LGP). Supernatant (about 5 mL) was transferred to 14-mL centrifuge tubes (Falcon 352059), and 5 mL of 3 M NaOAc (pH 5.2) was added. After mixing gently, 20 mL of isopropanol was added to precipitate DNA, which was then removed by pipette tip, washed in 1 mL of 70% ethanol, gently mixed, and centrifuged for 5 min at 8000 rpm. Liquid was removed and pellets were allowed to air-dry before addition of 500 mL TE (10 mM Tris-HCl, 1 mM EDTA, pH 8.0).

To remove RNA, 5 uL Ribonuclease A solution (1 mg/mL [Thermo Scientific Cat # AB-0549]) was added was added to 250 mL of DNA. Solutions were incubated at 37°C for 20 min then transferred to ice. DNA was precipitated by adding 500 uL of cold Ethanol (100%) and 100 uL of 5 M NH_4_Ac. Tubes were centrifuged at 13,000 rpms for 5 min, followed by removal of supernatant by pipetting. Pellets were washed with 500 uL of 70% ethanol, and centrifuged at 13,000 rpm for 5 min. Supernatant was removed and pellets were allowed to dry before re-suspension in 100 uL TE (10 mM Tris-HCl, 1 mM EDTA, pH 8.0).

### Sequencing library construction

Libraries for sequencing were prepared for each of the 24 pooled DNA samples as described in detail by McCarty et al. (2013b). Briefly, samples were sheared by sonication and size selected for an average size of 1 kb. Blunt end ligation was used to add double stranded tiB (Roche Inc., GS FLX Titanium General Library Preparation Manual), that would serve as priming targets for PCR. To amplify Mu-flanking sequences, we used a touchdown, two-step PCR reaction using the Mu TIR-specific primer, TIR6 (Settles et al., 2007; Supplemental Figure 2), and the adapter-specific primer, tiB (McCarty et al., 2013b; Supplemental Figure 2). Two additional rounds of nested PCR were used to incorporate 4-base barcodes and Illumina sequencing adaptors (see McCarty et al., 2013b). Libraries were pooled and concentrated using a concentrator column to achieve a final concentration of over 10 nM DNA. The combined library was sequenced in a single lane of an Illumina HiSeq II with unidirectional, 100-base sequencing.

### Bioinformatic analysis

Sequencing reads were screened for quality and trimmed as in McCarty et al. (2013b). Screens included analysis of overall sequencing quality, presence of a valid barcode, and inclusion of a complete TIR sequence. After barcode identifications were appended to the each read, the adapter and TIR sequences were trimmed. The remaining quality flanking sequences were used for precise mapping to the B73 reference genome by BLASTN (Altschul et al., 1990). Output from the alignment analyses were parsed into a custom database constructed using the Java Collections framework, described in McCarty et al. (2013b). The database output included chromosome positions and normalized read numbers for insertions identified in each of the 24 barcoded libraries (Supplemental Table 1). Normalization followed established protocols (McCarty et al., 2013b) to weight each sublibrary equally.

**Figure 2:**
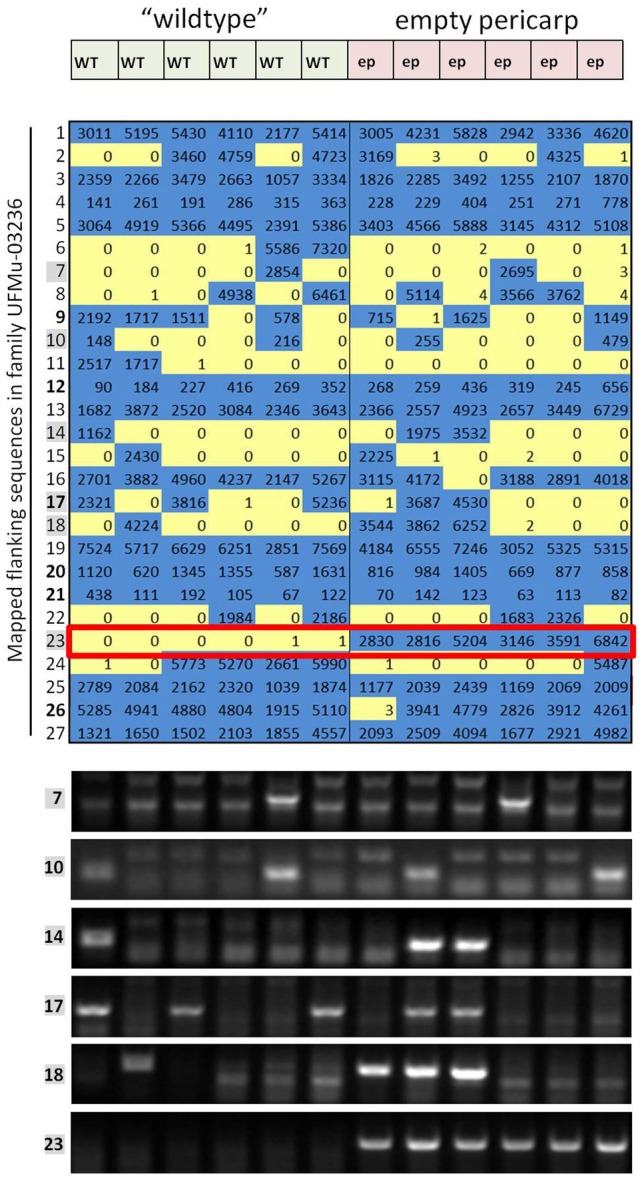
**Expanded results for one of the families in the forward-genetics Mu-seq grid**. Sequencing reads obtained for pool-7, corresponding to family UFMu-03236, are organized here based on insertion site (chromosome and location) and compared to phenotypic data to test co-segregation. In this example, normal ears made up the left half of the grid (pools A through F) and mutant ears were arranged on the right side of the grid (pools G through L, see Figure 1B). Of the 27 flanking sequences identified in pool 7 (assigned to family UFMu-03236), only number 23 showed co-segregation with the empty-pericarp phenotype (outlined in red). To check the consistency and reliability of the sequencing approach, including assignment of map locations, DNA from the individual plants included in the grid for this family was analyzed by PCR for the presence/absence of six of the segregating insertions (indicated by gray boxes). Results were consistent for PCR and sequencing.

A cut-off of 50 sequencing reads for an individual sample was used to call the presence of an insertion. The forward-genetic Mu-seq grid design allowed the presence or absence of each insertion to be resolved at the individual-plant level. Insertion locations and numbers of sequence reads flanking each of their sites were tabulated and arranged to visualize the genotypic status of each individual (Figure 2; Supplemental Table 1). The presence or absence of each insertion was compared to the distribution of mutant phenotypes in each family to test for co-segregation between them.

### Validation of co-segregations using PCR

To further test such instances of co-segregation, larger families segregating for the mutant phenotype of interest were genotyped by PCR using gene-specific and Mu-TIR-specific primers. For each of the seven insertions found to co-segregate with a mutant phenotype, PCR primers were designed that flanked the insertion site (Fwd primer upstream of insertion and Rev primer downstream). Primers were designed with melting temperatures between 63 and 65°C using web-based tools available from Integrated DNA Technologies (idtdna.com). Primer sequences are provided in Supplemental Figure 2. Gene-specific primers were used to detect presence of a normal gene copy, whereas one gene-specific primer was used in combination with a Mu-TIR-specific primer (TIR6) to detect the presence of an insertion. Reactions were run in an Eppendorf Mastercycler ProS thermocycler with the following parameters: 94°C for 5 min, then 40 cycles at 94°C for 45 s, 60°C for 90 s, 72°C for 100 s, then 72°C for 7 min. Products were separated on 1% agarose gels to visualize and test for of co-segregation of mutant phenotypes with the presence of expected, amplified DNA fragments.

### Localization predictions

Putative subcellular localizations were estimated using the web-based Target-P peptide prediction program (Emanuelsson et al., 2000, 2007). Predicted amino acid sequences for each putative, causal gene were obtained from maizesequence.org and analyzed using TargetP criteria for plant proteins with all parameters at their default settings. TargetP scores above.700 were considered predictive of organellar targeting.

## Results

To explore the potential of using forward-genetic Mu-seq for systematic analysis of maize mutants, we selected 12 families with visible-seed phenotypes identified in a screen of the UniformMu transposon population. We opted to use seed mutants due to our long-standing interest in kernel development (McCarty et al., 1991; McCarty, 1995; Andersen et al., 2002; Koch, 2004), the importance of these structures to humankind (Klopfenstein et al., 2012; Ray et al., 2012), and also because seed mutants represent a large, genetically-complex, phenotypic class. Our first step in developing a forward-genetic strategy was to implement a multi-generation, genetic pipeline that (1) tested whether phenotypes were recessive and heritable, (2) established segregating, backcross families that were suitable for linkage analysis, and (3) provided materials for phenotypic investigation and classification.

### Genetic strategy and sequencing grid design

The 12 families selected for analysis in the present study were characterized using the genetic strategy outlined in Figure 1A. This included selfing each putative heterozygous mutant plant to test for heritable phenotypes and concurrently back-crossing these individuals to W22-inbred females. Where phenotypes were heritable, F1 progeny of the backcrosses were selfed and the resulting ears were scored as segregating or non-segregating relative to the mutant phenotypes. For each mutant family, DNA from mothers of six segregating ears (heterozygous individuals) and six normal ears (homozygous individuals) were selected for inclusion in a forward-genetic Mu-seq grid. Balanced numbers of heterozygous and homozygous-normal individuals were not essential for this approach, therefore in cases where ears of either class (mutant or normal) were limited in number, then individuals from the other class were used in the grid (Figure 1B). The resulting 12-by-12 array of DNA from individuals bearing ears is diagramed in Figure 1B. The grid design was arranged to simplify interpretation of resulting sequence data by grouping mutant and non-segregating ears on the left or right side of the grid, alternating by family. Leaf samples had been collected from the parent plant of each ear and were pooled as shown in Figure 1B. The DNA for analyses was extracted from each of these 24 pools. Resulting DNA samples were each assigned a unique barcode (attached during construction of sequencing libraries) and sequenced together in a single Illumina flow cell (McCarty et al., 2013b).

### Analysis of sequencing grid results

In total, 341 unique, genic insertions (as determined by greater than 100 normalized sequencing read counts in the family-specific pool fraction) were identified and mapped in the twelve UniformMu families included in this forward-genetic Mu-seq grid (Table 1). Out of these, 290 insertions were present in multiple plants and thus confirmed as germinal mutations. The remaining 51 insertions were identified in single plants among twelve individuals within single families. The prevalence of rare insertions in one family (UFMu-04889, with 48 of 115 total insertions) is consistent with the presence of residual transposase activity in this line. Although genetic selection is used to limit transposase activity in plants prior to Mu-seq analysis, residual MuDR-activity is detected in about 1% of UniformMu lines (McCarty et al., 2013b). Because of the deep-sequence coverage obtained by forward-genetic Mu-seq, such single-plant insertions (whether from germinal or somatic transposition events) can be readily distinguished from segregating germinal insertions as above.

**Table 1:** **Sequencing results from forward-genetics Mu-seq grid**.

**Pool**	**Family**	**Phenotype**	**Genic insertions**	**Segregating insertions**	**Single-plant insertions**	**Positive co-segregation**
1	UFMu-00469	emb	33	33	0	1
2	UFMu-04413	ep	26	26	0	0
3	UFMu-04889	smk/emb	115	67	48	1
4	UFMu-03275	dek/emb	11	10	1	1
5	UFMu-00117	smk/emb	32	32	0	1
6	UFMu-00006	emb	5	5	0	0
7	UFMu-03236	ep	27	27	0	1
8	UFMu-01057	dek	7	7	0	0
9	UFMu-00298	ep	26	26	0	1
10	UFMu-05297	smk	8	8	0	1
11	UFMu-03318	ep	4	4	0	0
12	UFMu-01240	dek	47	45	2	0
			341	290	51	7

Phenotypes and numbers of genic Mu insertions are shown for the 12 families included in the sequencing grid. Segregating insertions indicate that an insertion was present in multiple individuals in a family. Single-plant insertions are those which appeared in one plant only, and suggest low levels of somatic transposition. Positive co-segregation indicates that an insertion was present in all mutant individuals and absent in all non-mutant individuals within a family. Seed phenotype abbreviations: embryo lethal (emb), empty pericarp (ep), small kernel (smk), defective kernel (dek).

Results from this forward-genetic Mu-seq grid (and others arranged as in Figure 1) can be readily interpreted as in Figure 2. Sequencing reads are parsed by barcode, trimmed, aligned to the maize reference genome, and assigned map locations as described in McCarty et al. (2013b). The process is further streamlined using custom, Java-based programs (McCarty et al., 2013b). During this process, each sequence is anchored to a specific genomic location and assigned a unique identifier. Numbers of reads at each map location are tabulated in sortable tables that are analyzed for co-segregation of a particular insertion position with a phenotype-of-interest. All sequencing reads obtained in pool #1, for example, represent Mu-flanking sequences from family #1 (see Figure 2). Where those same reads appear in pools A through L, they correspond to individual plants in family #1 that each carry the Mu insertions indicated.

To further test sequencing results from the forward-genetic Mu-seq grid, selected insertions were examined by PCR using DNA from individual leaf samples (family UFMu-03236, Figure 2). Primers were designed to amplify DNA flanking putative insertion sites and used together with a Mu-TIR-specific primer to amplify products if Mu insertions were present at the predicted location. In each of the instances tested, PCR and sequencing results agreed (Figure 2), demonstrating the accuracy and reliability of the approach.

For results to demonstrate a “positive co-segregation,” an exact correspondence was required between the presence or absence of a particular insertion and the presence or absence of a given phenotype. Such co-segregation was observed for seven of the twelve families tested. Another family showed close, but imperfect, linkage between an insertion and a phenotype. Further analysis of this family showed one individual that did not produce mutant seeds despite carrying an insertion that otherwise co-segregated with this phenotype (in family UFMu-01057, pool 8). In this case, the absence of tight linkage (and thus negation of the candidate gene) was confirmed by analysis of progeny in a larger segregating family (Supplemental Figure 1), further validating the efficacy and accuracy of forward-genetic Mu-seq.

### Identification of putative causal gene mutations

**Figure 3:**
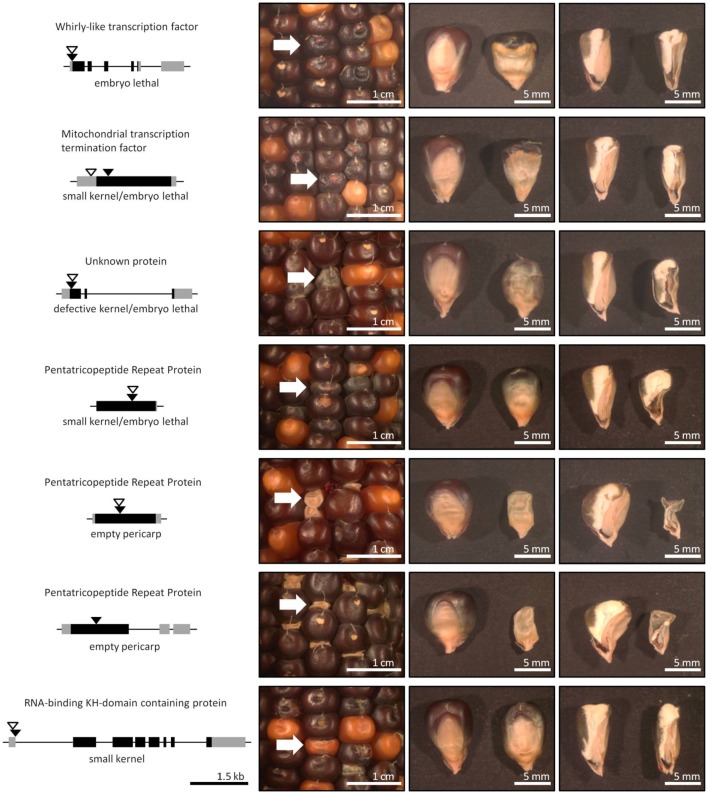
**Gene mutations that co-segregate with seed phenotypes**. The seven, positive, co-segregation results from the forward-genetics Mu-seq grid corresponded to insertions in seven maize genes. The genes are diagrammed on the left, with Mu insertion sites identified in the sequencing grid indicated by black triangles, coding sequence by black bars, and untranslated regions by gray bars. Black-bordered, open triangles indicate positions of additional alleles available in other UniformMu lines. Six of the seven insertion sites were in exons, with the exception being an insertion in the first intron of a predicted RNA-binding protein that co-segregated with a defective kernel phenotype (bottom panels). The phenotypes that co-segregate with each insertion are represented in the images to the right, showing on-ear appearance, embryo faces of mature kernels, and vertical, saggital sections of mature kernels (with normal seeds to the left and mutant seeds to the right). Representative normal and mutant kernels were selected for imaging. White arrows indicate mutant kernels.

The seven insertion sites that co-segregated perfectly with kernel phenotypes were analyzed for proximity to annotated maize genes (B73 reference v2, filtered gene set). In each instance, the Mu insertion was in or near the coding sequence associated with a gene (Table 2, Figure 3). Of these, four showed embryo-lethal phenotypes that co-segregated with Mu insertions in specific genes: one coding for the Whirly1 transcription factor, one for a putative mitochondrial transcription termination factor, one for an unknown protein, and one for a putative pentatricopeptide repeat family protein (PPR). Two of the empty-pericarp phenotypes co-segregated with insertions in different, putative PPR genes. Finally, a small-kernel phenotype co-segregated with an insertion in a predicted RNA-binding, KH-domain-containing gene. Six of the seven insertions are in putative exons, with the only exception being an insertion just downstream of the 5'-UTR in the first intron of an RNA-binding KH-domain-containing gene (Table 2, Figure 3).

**Table 2:** **Putative causal genes identified by forward-genetic Mu-seq**.

**Family**	**Gene**	**Putative annotation**	**Insertion location**	**Position**
UFMu-00469	GRMZM2G155662	Whirly1 transcription factor	Chr 6—71,621,420	Exon 1 UTR
UFMu-04889	GRMZM2G061542	Mitochondrial transcription termination factor	Chr 1—191,063,545	Exon 1 CDS
UFMu-03275	GRMZM2G084429	Unknown protein	Chr 8—133,438,934	Exon 1 CDS
UFMu-00117	GRMZM2G308189	Pentatricopeptide repeat (PPR-1)	Chr 5—181,583,967	Exon 1 CDS
UFMu-03236	GRMZM2G069078	Pentatricopeptide repeat (PPR-2)	Chr 8—170,866,001	Exon 1 CDS
UFMu-00298	GRMZM2G035664	Pentatricopeptide repeat (PPR-3)	Chr 1—240,273,866	Exon 1 CDS
UFMu-05297	GRMZM2G143568	RNA-binding KH domain-containing protein	Chr 10—61,378,846	Intron 1

Co-segregation between phenotype and a Mu insertion was observed for 7 of the 12 UniformMu families included in the forward-genetic Mu-seq grid. Specific Mu insertions that co-segregated with the phenotypes were present in putative genes from the maize, filtered gene set (v2). The candidate genes were assigned annotations by sequence comparison to Arabidopsis and rice databases. Transposon insertion sites within the genome and within each gene are indicated in columns 4 and 5. Phenotypes from the other 5 families included in the grid did not show a Mu insertion co-segregating.

### Validation of co-segregation results

Locus-specific PCR was used to further validate positive co-segregations obtained from grid sequencing. Individuals from larger F2 families (of 20 or more individuals) segregating for the phenotypes-of-interest were scored as mutant or non-mutant and genotyped. Gene-specific primers flanking the insertion site were used to test for the presence of a wildtype copy of the gene, and one gene-specific primer, together with a Mu-TIR-specific primer were used to test for the presence of an insertion in the gene. Co-segregation was confirmed in all six instances where Mu-insertion sites were amenable to PCR (Table 3, Supplemental Figure 1). Attempts to amplify the 7th putative causal insertion (that in the RNA-binding KH-domain-containing gene) using multiple primer combinations were unsuccessful. Map distances were calculated with 95% confidence based on the total number of individuals genotyped, including both PCR- and sequence-based approaches. The formula used for calculating map distances was: distance in centimorgans $=\left(1-0.05^{\frac{1}{N}}\right)*100$, where *N* is the number of individuals genotyped.

**Table 3:** **Genetic analyses of progeny**.

**Putative annotation**	**Phenotype**	**Positive co-segregation?**	**Individuals genotyped**	**Map distance**
Whirly1 transcription Factor	emb	Yes	21/21	13.3 cM
Mitochondrial transcription termination factor	smk/emb	Yes	28/28	10.1 cM
Unknown protein	dek/emb	Yes	35/35	8 cM
Pentatricopeptide repeat (PPR-1)	smk/emb	Yes	21/21	13.3 cM
Pentatricopeptide repeat (PPR-2)	ep	Yes	35/35	8.2 cM
Pentatricopeptide repeat (PPR-3)	ep	Yes	29/29	9.9 cM
RNA-binding KH domain-containing protein	smk	PCR recalcitrant	12/12	22.1 cM

Individual PCR used to further validate co-segregation of phenotypes with insertions identified in the forward-genetics Mu-seq grid within segregating F2 families. Positive co-segregation indicates that the putative causal insertions showed perfect co-segregation in all individuals tested. Map distances were estimated based on the number of individuals genotyped either by sequencing or PCR, and is reported at a 95% confidence level. Seed phenotype abbreviations: embryo lethal (emb), empty pericarp (ep), small kernel (smk), and defective kernel (dek).

Additional mutant alleles for each of the seven candidate genes were sought by searching the UniformMu database (available at MaizeGDB.org). Second mutant alleles with insertions in different sites were available for six of the seven target genes (Figure 3; Table 4). For each of those six genes, a UniformMu line was selected that carried a second mutant allele. The lines chosen were prioritized for insertions in coding vs. non-coding sequences. These UniformMu lines were grown, self pollinated, and ears were examined for seed phenotypes. Locus-specific PCR was used to test for specific insertions and for their co-segregation with identified seed phenotypes (Table 4). Additional alleles from the Whirly1 transcription factor and two of the PPR genes showed co-segregation with like-phenotypes, confirming causality for mutations in these genes and the associated phenotypes. The additional allele in the mitochondrial transcription termination factor was not amplified by PCR, so co-segregation with the expected phenotype could not be confirmed in this instance. Insertions in the unknown protein-coding gene and the RNA-binding KH domain-containing genes were identified, but visible phenotypes were not evident (Table 4).

**Table 4:** **Genetic analyses of additional alleles from UniformMu**.

**Putative annotation**	**UniformMu family**	**Position**	**Phenotype**	**Co-segregates?**
Whirly1 transcription factor	UFMu-06220	Exon 1 UTR	emb	Yes
Mitochondrial transcription termination factor	UFMu-08145	Exon 1 UTR	smk/emb	PCR recalcitrant
Unknown protein	UFMu-01783	Exon 1 CDS	none	N/A
Pentatricopeptide repeat (PPR-1)	UFMu-03453	Exon 1 CDS	smk/emb	Yes
Pentatricopeptide repeat (PPR-2)	UFMu-03459	Exon 1 CDS	ep	Yes
Pentatricopeptide repeat (PPR-3)	None available	N/A	N/A	N/A
RNA-binding KH domain-containing protein	UFMu-06477	Exon 1 UTR	none	N/A

The UniformMu database was searched for Mu insertions in the seven putative, causal genes identified in the forward-genetic Mu-seq grid. One allele was chosen for each gene (when available). Progeny from these lines were phenotyped and genotyped using PCR to test for co-segregation. Phenotype abbreviations: embryo lethal (emb), small kernel (smk), defective kernel (dek), empty pericarp (ep).

Mutations associated with this sample of kernel phenotypes predominated in genes predicted to mediate nuclear control over organellar-gene processing, thus highlighting the importance of this process during seed development. Transit-peptide analysis indicated that four of the predicted proteins are targeted to mitochondria and one to plastids (Table 5). Notably, each of the three putative PPR genes are predicted to encode mitochondrial-targeted proteins. Despite this similarity, all are linked with distinctive kernel phenotypes, ranging from empty pericarp to embryo lethality (Figure 3).

**Table 5:** **Predicted localizations of proteins encoded by the putative, causal genes carrying Mu insertions**.

**Putative annotation**	**Predicted localization**	**TargetP score**	**Phenotype**
Whirly1 transcription factor	Plastid	0.874	emb
Mitochondrial transcription termination factor	Mitochondria	0.912	smk/emb
Unknown protein	?		dek/emb
Pentatricopeptide repeat (PPR-1)	Mitochondria	0.720	smk/emb
Pentatricopeptide repeat (PPR-2)	Mitochondira	0.857	ep
Pentatricopeptide repeat (PPR-3)	Mitochondria	0.832	ep
RNA-binding KH domain-containing protein	?		smk

Signal-peptide analysis for the seven, putative, causal genes for seed phenotypes were examined using the web-based TargetP utility. Scores represent confidence of the predicted localization. Values over 0.7 were considered strongly predictive. A “?” indicates no localization prediction obtained. Phenotype abbreviations: embryo lethal (emb), small kernel (smk), defective kernel (dek), empty pericarp (ep).

## Discussion

Here we present a new set of putative causal genes underlying kernel phenotypes in maize and introduce the strategy and protocol used to obtain them. To achieve reliable, efficient, co-segregation analyses of segregating mutant families, we developed “forward-genetic Mu-seq.” The speed and efficacy of forward-genetic Mu-seq were enabled by an approach that included existing technology, redesigned for high-throughput, phenotype-to-genotype analyses. This was used together with a streamlined genetic pipeline for establishing families that segregated for mutations of interest, and a gridding design for integrated analysis of genotypes and phenotypes. Candidate mutations were successfully obtained for seven of twelve maize mutants examined concurrently in a single experiment. This approach is particularly advantageous for analysis of the high-copy Mutator transposon system in maize, and may be adapted to other multi-copy transposon systems with conserved TIR sequences. Conventional approaches for genetic analysis of Mutator lines, which may contain in excess of 100 unique transposon insertions per individual, typically entail multiple generations of backcrossing and selection to reduce the number of Mu elements in plants analyzed. By enabling simultaneous tracking of all insertions in a population, forward-genetic Mu-seq allows testing of correlations between genotype and phenotype without extensive backcrossing. Another key feature of this protocol is that genetic analyses of multiple insertions can be performed without needing a specific genotyping assay for each mutation. The development and validation of gene-specific PCR assays for genotyping is frequently time-consuming and too-often frustrating. Moreover, we find that a Mu-seq approach detects Mu insertions that are recalcitrant to classical PCR methods (Table 3; Supplemental Figure 1). Relieving this constraint is especially important for emerging, large-scale, functional genomics applications, such as testing knockouts in multiple candidate genes for QTLs that may require genotyping multiple insertions of interest in many hundreds of individuals. Also, forward-genetics Mu-seq will become progressively more cost-effective with continuing advances in sequencing technology.

Forward-genetics Mu-seq is particularly well-suited to co-segregation analyses in studies of recessive seed mutants that can be observed on mature, self-pollinated ears. Presence of such kernel mutants on an ear indicate a heterozygous parent carrying a single copy of the mutant gene (as two copies yield seed lethality, making recovery of homozygous plants impossible), whereas lack of such kernels indicate a wildtype parent. Such plants (or progeny from a back-cross such as shown in Figure 1A) are ideal for constructing forward-genetic Mu-seq grids since presence or absence of a given Mu insertion can be readily scored from sequence data to test for co-segregation. Non-lethal can also be pursued using this approach. In such instances, it would be ideal to grow F2 progeny to observe phenotypes prior to gridding, allowing heterozygous, homozygous, and wildtype parents to be scored (similar to scoring an ear for a segregating seed phenotype). In some instances it may even be possible to distinguish homozygous from heterozygous individuals in a sequencing grid. Theoretically, insertions should yield twice the number of sequencing reads when homozygous vs. heterozygous. While there is a high degree of variability in read number from insertion to insertion, we have observed good consistency in read number for a given insertion from sample to sample (see Figure 2; McCarty et al., 2013b). We suggest that the variability occurs because each insertion is amplified to different, yet often-repeatable degrees during construction of sequencing libraries depending on the insertion locale and flanking-sequence composition. At present, however, variability in read number precludes an effective, repeatable delineation of homozygous and heterozygous insertions.

Strong candidate genes are identified by the relationships between Mu insertions and closely-linked phenotypes reported here. Second alleles or complementation experiments are needed for confirmation. Analysis of additional mutant alleles confirmed associations between mutations in the Whirly1 transcription factor and two of the PPR genes with seed lethal phenotypes (Table 4). Causality is yet to be determined between the identified Mu insertions and the phenotypes for the other four candidate genes. Alternative UniformMu alleles were not available for the third PPR gene. In the case of the mitochondrial transcription factor gene, the second allele segregated for the expected embryo lethal phenotype, but we were unable to amplify the target insertion using PCR. For the RNA-binding KH domain-containing gene and the Unknown protein-coding gene, second alleles did not produce see phenotypes (Table 4), despite being detected by PCR. The initially-identified insertions may be linked with the causal mutation, but not directly responsible for the phenotypes. Alternatively, insertion positions at different sites in a gene can have contrasting effects on gene transcript accumulation (Greene et al., 1994; Girard and Freeling, 2000; Settles et al., 2001; Cui et al., 2003). In particular, we have found that insertions in 5' UTRs are often ineffective in conferring a dysfunctional mutation (unpublished results). Such insertions can sometimes be spliced as introns or ignored in untranslated regions.

Mutant alleles for one of the seven candidate genes have been previously described, confirming that the insertion identified in the present study was indeed causal. Zhang et al. (2013) showed that mutations to the Whirly1 transcription factor (allelic to the one revealed here) lead to embryo-defective phenotypes. Interestingly, Prikyryl et al. (2008) had previously described two mutations in the same Whirly1 transcription factor that conferred albino-seedling phenotypes, but not defective embryos. Background effects can markedly alter expression of phenotypes in maize (Lee et al., 1994; Hunter et al., 2012), and indeed, Zhang et al. (2013) demonstrated this by introgressing UniformMu alleles of the Whirly1 transcription factor into non-W22 inbreds, where albino seedlings were observed.

One particularly striking finding of the forward-genetic Mu-seq grid analyzed here was the predominance of mutants in genes coding for predicted organellar-targeted proteins. The forward-genetic Mu-seq approach can be particularly useful when genotype-to-phenotype investigations focus on dissecting developmental pathways. Selecting like-phenotypes from a collection of mutants and genotyping them using this strategy enables targeted analysis of whichever pathways affect the phenotype of interest. The grid sequenced here was directed toward seed phenotypes, and results indicate a pivotal role for mitochondrial RNA-editing in kernel development. Four of the seven candidate genes are predicted to modulate that process at some level (Table 5). This finding is analogous to that of a growing number of studies pointing to prominent roles of organellar function in embryogenesis and seed development (McElver et al., 2001; Schmitz-Linneweber and Small, 2008; Myouga et al., 2010; Bryant et al., 2011; Steinnebrunner et al., 2011; Holdorf et al., 2012; Benz et al., 2013).

Pentatricopeptide repeat-containing proteins, in particular, have been implicated in seed-development phenotypes of both Arabidopsis (Tzafrir et al., 2003; Lurin et al., 2004; Tzafrir et al., 2004; Cushing et al., 2005; Falcon de Longevialle et al., 2007; Lu et al., 2011) and maize (Gutiérrez-Marcos et al., 2007; Manavski et al., 2012; Sosso et al., 2012a,b; Liu et al., 2013). In subsequent, forward-genetic Mu-seq grids that targeted defective-kernel mutants, we have identified an additional 12 insertions in PPR-containing genes as putative, causal mutations (unpublished data). Collectively, these mutations in 15 PPR-containing genes represent approximately half of the total, putative, causal-genes we have identified in our research on seed mutants using this approach. The outcome is clearly consistent with the importance of PPR proteins in maize kernel development.

The number of unrecovered mutations in the forward-genetics Mu-seq described in the present paper was higher than initially predicted, with five of twelve mutants not showing a co-segregating gene mutation. One of the possible explanations is that some of the mutations are not caused by Mu transposons. Deletion alleles have been obtained from Mutator-based resource populations, including UniformMu (Robertson et al., 1994; Bortiri et al., 2006), though their prominence has not been thoroughly examined. Other possibilities may reflect limitations of our present approach. Mu-seq libraries are currently prepared using primers that bind to the conserved TIRs of the canonical Mu transposons, Mu1 through Mu9 and Mu13 through Mu19 (Dietrich et al., 2002; Tan et al., 2011; McCarty et al., 2013b). The Mutator family includes more diverse members (Mu10, Mu11, and Mu12) that are known to be active at some level Mutator-derived stocks (Dietrich et al., 2002). Molecular tools designed to recognize the canonical Mu transposons do not recognize the TIRs from these more diverse Mu transposons. Some of the unrecovered mutants from this grid may be caused by non-canonical elements. Another potential limitation may lie in the current, data-analysis pipeline. Mu-flanking sequences that do not map to the B73 filtered gene set are excluded from further analysis and are not used for comparison to phenotypes for co-segregation analysis. However, as noted above, this has been less than 3% of insertions examined thus far. We are continually improving our ability to detect Mu insertion sites and are pursuing non-tractable mutants to understand their extent and role in the UniformMu population. Forward-genetic Mu-seq nonetheless offers a successful means of capturing putative causal genes for about 50% of visible mutants from the UniformMu resource. A similar level of efficacy has been evident in subsequent, forward-genetic Mu-seq grids (unpublished data).

A two-dimensional grid design (as used in the present study) would typically be the preferred strategy in forward genetic Mu-seq, because it enables a more thorough analysis than single-dimension sequencing alone. Single-dimension strategies would theoretically be adequate where previous work had identified most of the new Mu insertions in a given line. This is the case for UniformMu material since Mu insertions in these families were mapped during construction of the public resource. In high-throughput analyses of such material, where speed or cost savings are paramount, co-segregation using single-dimension sequencing may be desirable. However, results will rely on causal insertions having already been assigned to a family. In the grid shown here, sequencing pools A through H would have identified only 6 of the 7, putative, causal insertions. This is because the 7th had not yet been identified in the UniformMu lines tested (possibly due to limited presence among sibling material sampled at the time of resource generation), so could not be assigned to the correct family without sequencing pools 1 through 12. While single-dimensional sequencing would have yielded good results, a two-dimensional grid design gives a greater level of confidence with little additional effort or cost (since all of the sequencing is done in a single flow cell). Our current system of 4-base barcodes enables simultaneous genotyping of up to 1024 individuals in a two-dimension, 32 × 32 grid. Although recovery of reads from individual insertions can vary up to 800-fold depending on genome context and other factors that are not readily simulated (McCarty et al., 2013b), our empirical tests indicate that a single HiSeqII lane typically provides adequate coverage of grids of at least 25 × 25 (625 individuals).

Forward-genetic Mu-seq for fast, efficient genotyping of Mutator-derived maize lines vastly increases the throughput of genetic analyses. The small-scale study presented here highlights the power and simplicity of the technology and demonstrates the effectiveness of the strategy for dissecting specific developmental pathways. By analyzing defective kernel mutants using a forward-genetic Mu-seq strategy, a clearer picture of the genes and processes important for kernel development is emerging. Mitochondrial and plastidial gene transcription and processing, under the control of nuclear-encoded proteins, clearly play a predominant role. Here we see a prominent degree of non-redundancy for these important genes and the relative abundance of organelle-targeted proteins whose mutations affect kernel development.

## Author contributions

Charles T. Hunter, Masaharu Suzuki, Donald R. McCarty, and Karen E. Koch each contributed to the experimental design and implementation of the work. Charles T. Hunter, Jonathan Saunders, Shan Wu, and Alexander Tasi were each central to conducting the experiments and acquiring the data presented in the work. Charles T. Hunter and Karen E. Koch were principally responsible for drafting the final manuscript. All authors provided intellectual content and contributed to manuscript revisions. All authors provided final approval of the manuscript. All authors agree to be accountable for all aspects of the work, including ensuring the accuracy and integrity of the work.

### Conflict of interest statement

The authors declare that the research was conducted in the absence of any commercial or financial relationships that could be construed as a potential conflict of interest.

## Supplementary material

Supplemental Figure 1 **Follow-up co-segregation PCR results**. Co-segregation of phenotypes with insertions identified in the sequencing grid was tested in the following generation. Selfed ears from segregating families were examined for phenotypes, and DNA from parent plants was used for PCR to test for co-segregation. Gene-specific primers flanking the insertion site were used to test for the presence of a wildtype copy of the gene (F plus R), and one gene-specific primer along with a Mu-TIR-specific primer were used to test for the presence of an insertion in the gene (F plus TIR6). Mutants were present as heterozygotes, so both wildtype and mutant bands were observed. The gene model shown indicates primer locations for an insertion in the unknown protein for which co-segregation of a Mu insertion with a defective kernel phenotype was tested. The Mu insertion site is indicated by a black triangle, primers by small black arrows, coding sequence by black bars, and untranslated regions by gray bars. Co-segregation between seed phenotypes and candidate Mu insertions was observed for all genes, with the exception of the putative sugar-phosphate translocator (bottom right), which showed no correlation between the presence of a defective kernel phenotype and the Mu insertion of interest (and was one off from co-segregating in the sequencing grid). A “?” represents an ear for which the phenotype was ambiguous. Seed phenotype abbreviations: embryo lethal (emb), empty pericarp (ep), small kernel (smk), defective kernel (dek).

Supplemental Figure 2 **PCR primer sequences and annealing temperatures**. Melting temperatures were estimated using the web-based tool from Integrated DNA Technologies (idtdna.com), with all parameters set at default. ^a^Primer was used in genotyping to confirm co-segregation in larger F2 families. ^b^Primer was used in Pool 7 validation experiments. ^c^Primer was used in sequencing library preparation.

Supplemental Table 1 **List of Mu insertion locations identified**. Insertion positions are presented organized by family of origin. Normalized read counts are shown for pools A through L, corresponding to the 12 plants genotyped for each family. Cells highlighted blue indicate presence of a given insertion (with 50 or more reads detected), and cells highlighted yellow indicate absence of a given insertion (with less than 50 reads detected).
