# Supplementary material for: Phenotype to genotype using forward-genetic Mu-seq for identification and functional classification of maize mutants
Source: Front Plant Sci. 2014 Jan 7;4:545. doi: 10.3389/fpls.2013.00545 (PMC3882665; doi:10.3389/fpls.2013.00545)
Supplement: Supplemental Figure 1 — Follow-up co-segregation PCR results. Co-segregation of phenotypes with insertions identified in the sequencing grid was tested in the following generation. Selfed ears from segregating families were examined for phenotypes, and DNA from parent plants was used for PCR to test for co-segregation. Gene-specific primers flanking the insertion site were used to test for the presence of a wildtype copy of the gene (F plus R), and one gene-specific primer along with a Mu-TIR-specific primer were used to test for the presence of an insertion in the gene (F plus TIR6). Mutants were present as heterozygotes, so both wildtype and mutant bands were observed. The gene model shown indicates primer locations for an insertion in the unknown protein for which co-segregation of a Mu insertion with a defective kernel phenotype was tested. The Mu insertion site is indicated by a black triangle, primers by small black arrows, coding sequence by black bars, and untranslated regions by gray bars. Co-segregation between seed phenotypes and candidate Mu insertions was observed for all genes, with the exception of the putative sugar-phosphate translocator (bottom right), which showed no correlation between the presence of a defective kernel phenotype and the Mu insertion of interest (and was one off from co-segregating in the sequencing grid). A “?” represents an ear for which the phenotype was ambiguous. Seed phenotype abbreviations: embryo lethal (emb), empty pericarp (ep), small kernel (smk), defective kernel (dek). [file Presentation1.PDF]

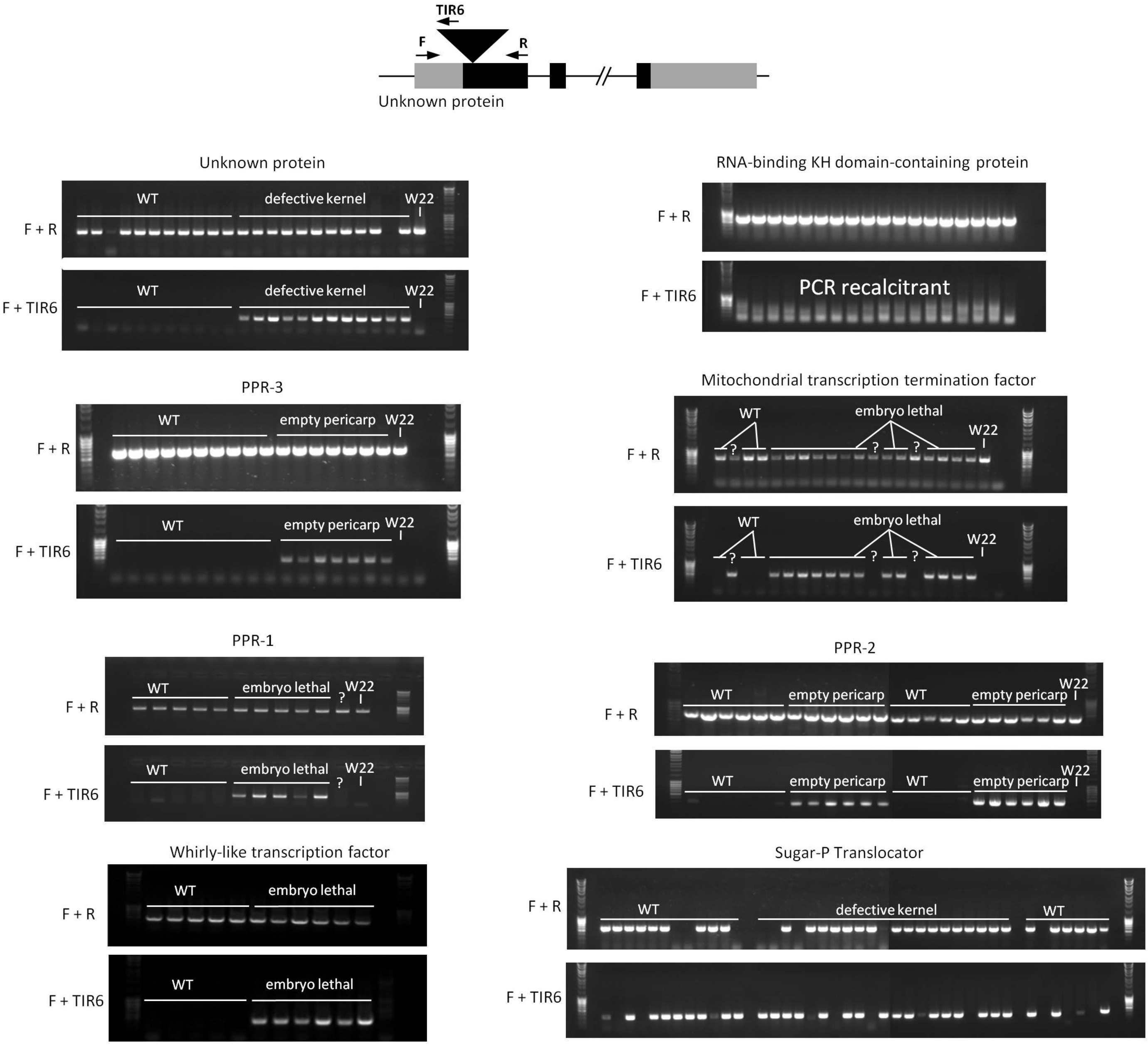

TIR6
F
R
Unknown protein
Unknown protein
F + R
WT
defective kernel
W22
F + TIR6
WT
defective kernel
W22
RNA-binding KH domain-containing protein
F + R
F + TIR6
PCR recalcitrant
PPR-3
F + R
WT
empty pericarp
W22
F + TIR6
WT
empty pericarp
W22
Mitochondrial transcription termination factor
F + R
WT
embryo lethal
W22
?
?
?
F + TIR6
WT
embryo lethal
W22
?
?
?
PPR-1
F + R
WT
embryo lethal
?
W22
F + TIR6
WT
embryo lethal
?
W22
PPR-2
F + R
WT
empty pericarp
WT
empty pericarp
W22
F + TIR6
WT
empty pericarp
WT
empty pericarp
W22
Whirly-like transcription factor
F + R
WT
embryo lethal
F + TIR6
WT
embryo lethal
Sugar-P Translocator
F + R
WT
defective kernel
WT
F + TIR6
