# Supplementary material for: Phenotype to genotype using forward-genetic Mu-seq for identification and functional classification of maize mutants
Source: Front Plant Sci. 2014 Jan 7;4:545. doi: 10.3389/fpls.2013.00545 (PMC3882665; doi:10.3389/fpls.2013.00545)
Supplement: Supplemental Figure 2 — PCR primer sequences and annealing temperatures. Melting temperatures were estimated using the web-based tool from Integrated DNA Technologies (idtdna.com), with all parameters set at default. aPrimer was used in genotyping to confirm co-segregation in larger F2 families. bPrimer was used in Pool 7 validation experiments. cPrimer was used in sequencing library preparation. [file Presentation2.PDF]

Gene	Primer Name	Primer Sequence (5` to 3`)	Tm (°C)
Whirly-like transcription factor	[a]WHRLY-F	GCA GCG AAG GCA GCG CTA CTG G	65.3
	[a]WHRLY-R	GCC GGT TCA CCG TCG GCC	65.1
Mitochondrial transcription termination factor	[a]Mito-F	TTC TGG GCT TTG AAG CCT TTG ATC CTC GCG	65.4
	[a]Mito-R	CTC GGT ACG GCT CAC CGT GTT CCA C	64.9
Unkown protein	[a]Unk-F	AGA TGA CGC AGA CGC AAA CCT TGT AGT CGA GC	65.7
	[a]Unk-R	TGC AGC AGC TTC TGG TGT CTA GAA GCT GG	65.0
PPR-1	[a]PPR1-F	AAG TCC TTG ATT CCA TGG GTT CTC AGG GC	63.5
	[a]PPR1-R	CCG ACC TTG CAG AGA CCA TGG ATC AGA AGG G	65.9
PPR-2	[a]PPR2-F	GAC GAG CCT TGC ACA GAG GGC C	64.8
	[a]PPR2-R	GGT ACA AGA ACG TGC AGG ACG CGG AG	64.9
PPR-3	[a]PPR3-F	GAG GCG CTC GAC AGG GCG ATT G	64.8
	[a]PPR3-R	GCA GCT GGG GGA ACA CCA CAG	65.1
RNA-binding KH domain-containing protein	[a]RNAbKH-F	GTC CAG TTC TCG GTC CAT CCA ACT CCC C	65.3
	[a]RNAbKH-R	CAC AGG ACA CTG GGA CTT GTC AAG GAG GG	65.1
Mutator TIR	[a,b,c]TIR6	AGA GAA GCC AAC GCC AWC GCC TCY ATT TCG TC	65.6 – 67.7
Chr 1: 105325442	[b]1:105325442-F	GTT GTG CAG GTC ACA TGT ACG CCA GCC	65.3
	[b]1:105325442-R	CGC TCC CTA CTC GGA GGC GG	64.5
Chr 2: 15128969	[b]2:15128969-F	GGC CGT CAA ACC CGC CGT C	64.6
	[b]2:15128969-R	GTA GGT GAG CTC CGG CTG CGG	64.8
Chr 3: 93414937	[b]3:93414937-F	GCA GCA GGC AGG ACG AGA GTG C	64.9
	[b]3:93414937-R	CGG CCT CCT TGA GGA CGG CC	65.5
Chr 6: 124881031	[b]6:124881031-F	GTG GTG CGC AGC GGT CCG	65.3
	[b]6:124881031-R	CTG GCC CGC CCG CAT TCA TCT C	65.2
Chr 7: 16272205	[b]7:16272205-F	CCT AAG CCT AGC CGA GCC GCC	64.7
	[b]7:16272205-R	GAC CGC GAC GAC TTG GCC GAC	65.3
Chr 7: 169216768	[b]7:169216768-F	GGC GCC ATG GCG TCG ACG	65.3
	[b]7:169216768-R	CGC TCC GTT CTC ATC CCG CTC TCA C	64.8
tiB	[c]tiB	CCTATCCCCTGTGTGCCTTGGCAGTCTCAG	66.3
Mu-Seq adapter primer (NNNN is barcode)	[c]Mu-Seq N	ACACGACGCTCTTCCGATCTNNNNCBCTCTTCKTCYATAATGGCAAT	64.6 – 71.8
A2-TiB adaptor primer	[c]A2 adapter	CAAGCAGAAGACGGCATACGAGATCGCCTTGGCAGTCTCAG	69.0
Illumina A1 sequencing adaptor primer 1	[c]SolfcA_Seq1.2	AATGATACGGCGACCACCGAGATCTACACTCTTTCCCTACACGACGCTCTTCCGA	70.8
Illumina A1 sequencing adaptor primer 2	[c]f SolfcB	CAAGCAGAAGACGGCATACGAGATC	59.3
